# Language specific listening of Japanese geminate consonants: a cross-linguistic study

**DOI:** 10.3389/fpsyg.2014.01422

**Published:** 2014-12-11

**Authors:** Makiko Sadakata, Mizuki Shingai, Simone Sulpizio, Alex Brandmeyer, Kaoru Sekiyama

**Affiliations:** ^1^Centre for Cognition, Donders Institute for Brain, Cognition and Behaviour, Radboud University NijmegenNijmegen, Netherlands; ^2^Institute for Logic, Language and Computation, University of AmsterdamAmsterdam, Netherlands; ^3^Division of Cognitive Psychology, Kumamoto UniversityKumamoto, Japan; ^4^Department of Psychology and Cognitive Science, University of TrentoRovereto, Italy; ^5^Fondazione ONLUS Marica De VincenziRovereto, Italy; ^6^Max Planck Institute for Cognitive and Brain SciencesLeipzig, Germany

**Keywords:** phonology, orthography, perception, cross-linguistic, geminate consonants

## Abstract

Various aspects of linguistic experience influence the way we segment, represent, and process speech signals. The Japanese phonetic and orthographic systems represent geminate consonants (double consonants, e.g., /ss/, /kk/) in a unique way compared to other languages: one abstract representation is used to characterize the first part of geminate consonants despite the acoustic difference between two distinct realizations of geminate consonants (silence in the case of e.g., stop consonants and elongation in the case of fricative consonants). The current study tests whether this discrepancy between abstract representations and acoustic realizations influences how native speakers of Japanese perceive geminate consonants. The experiments used pseudo words containing either the geminate consonant /ss/ or a manipulated version in which the first part was replaced by silence /_s/. The sound /_s/ is acoustically similar to /ss/, yet does not occur in everyday speech. Japanese listeners demonstrated a bias to group these two types into the same category while Italian and Dutch listeners distinguished them. The results thus confirmed that distinguishing fricative geminate consonants with silence from those with sustained frication is not crucial for Japanese native listening. Based on this observation, we propose that native speakers of Japanese tend to segment geminated consonants into two parts and that the first portion of fricative geminates is perceptually similar to a silent duration. This representation is compatible with both Japanese orthography and phonology. Unlike previous studies that were inconclusive in how native speakers segment geminate consonants, our study demonstrated a relatively strong effect of Japanese specific listening. Thus the current experimental methods may open up new lines of investigation into the relationship between development of phonological representation, orthography and speech perception.

## INTRODUCTION

Cross-linguistic studies of speech perception have unearthed language-specific differences in the perception and representation of basic speech sounds (Cutler, 2012). Language specific listening can be reflected by disagreements of phonemic categorical boundaries across language groups (e.g., /b/-/p/ continuum, Liberman et al., 1961; Brandmeyer et al., 2012), or by inter-language differences in the number of identified categories spanning a certain acoustic continuum (e.g., Japanese collapsing the /r/ – /l/ continuum into one single category, Bradlow et al., 1997). Selective sensitivity to language-specific acoustic information also influences the perception of non-linguistic auditory materials. For example, speakers of languages that include durational contrasts in their phonemic inventory are more sensitive to changes in duration in general (Pickett et al., 2000; Ylinen et al., 2005; Sadakata and Sekiyama, 2011). Another well-known example is the use of language-specific segmentation/representation strategies. Here, studies using the Japanese language have been able to make considerable contributions due to its use of a distinct segmentation unit. Japanese listeners usually exhibit different segmentation strategies than, e.g., native listeners of English and/or French, especially when tested with words including sounds that could constitute a separate unit (Otake et al., 1993, 1996; Cutler and Otake, 1994; Murty et al., 2007). Dupoux et al. (1999) and Dehaene-Lambertz et al. (2000) specifically highlighted the fact that Japanese phonotactics have a strong influence on speech perception; as coda consonants do not occur in Japanese (except for nasal consonants), Japanese listeners tend to perceive an illusory epenthetic vowel /u/ in a consonant cluster (e.g., /ebzo/ is often perceived as /ebuzo/ by Japanese listeners). The current study highlights another unique case in which Japanese native listeners exhibit a specific listening tendency.

The focus of this study is the perception of Japanese geminate consonants: double consonants such as /ss/ or /kk/. Geminate consonants are allophones of single consonants that form minimal pairs with them and which are lexically important. Geminate consonants also occur in many other languages such as Italian (Pickett et al., 2000; Bertinetto and Loporcaro, 2005; Tagliapietra and McQueen, 2010) and Finnish (Ylinen et al., 2005). The duration from the offset of a preceding vowel to the onset of a vowel immediately following the geminate consonants tends to be longer than it’s singleton counterpart. Such consonants are often referred as long (geminate) or short (singleton) consonants (Kingston et al., 2009; Hardison and Motohashi-Saigo, 2010; Tagliapietra and McQueen, 2010). As such, the durational information provided by these phonemes largely accounts for the acoustic characteristics of this contrast and provides a crucial perceptual cue for native listeners (Kingston et al., 2009; Amano and Hirata, 2010) among other covariate cues, such as patterns of fundamental frequency and loudness (see for example, Kawahara, 2006; Idemaru and Guion, 2008; Sato et al., 2012). Moreover, learning to perceive such contrasts is not easy for speakers of non-geminate languages, suggesting that language specific listening also influences to the perception of this contrast (Motohashi-Saigo and Hardison, 2009; Hardison and Motohashi-Saigo, 2010; Tajima et al., 2010; Sadakata and McQueen, 2013). It has been previously established that Japanese native speakers show an enhanced perceptual sensitivity for consonant timing (Sadakata and Sekiyama, 2011). Additionally, native listeners may utilize other language-specific features of Japanese when perceiving geminates consonants, such as abstract categorical representations. The motivation for this hypothesis comes from the fact that the representation of geminate consonants in Japanese phonology and orthography deviates considerably from other languages.

As mentioned above, it has been convincingly demonstrated that speech segmentation is greatly influenced by phonology (Otake et al., 1993, 1996; Cutler and Otake, 1994; Dupoux et al., 1999; Dehaene-Lambertz et al., 2000; Murty et al., 2007) and orthography (Seidenberg and Tanenhaus, 1979; Ziegler and Ferrand, 1998; Ventura et al., 2004; Pattamadilok et al., 2007; Rastle and Davis, 2008; Perre et al., 2009; Rastle et al., 2011). A number of computational models have been proposed to account for the influence of such top–down lexical effects on phonemic decisions during speech processing. For example, the TRACE model (McClelland and Elman, 1986) explains that this effect takes place as a result of continuous communication between the processing of bottom–up acoustic features and top–down lexical representations. Later computational models, such as Shortlist (Norris, 1994), Merge (Norris et al., 2000) and Shortlist B (Norris and McQueen, 2008), explain this effect in a purely feed-forward manner: once bottom–up features activate candidate representations at a higher level (e.g., input phonemes activate a list of candidate words), the lexical competition is restricted among these candidates. Despite their underlying differences, these computational models, together with the results of the previously mentioned experimental studies, suggest that speech processing is hierarchical in nature, and thus is subject to the influence of different types of linguistic representations.

**FIGURE 1:**
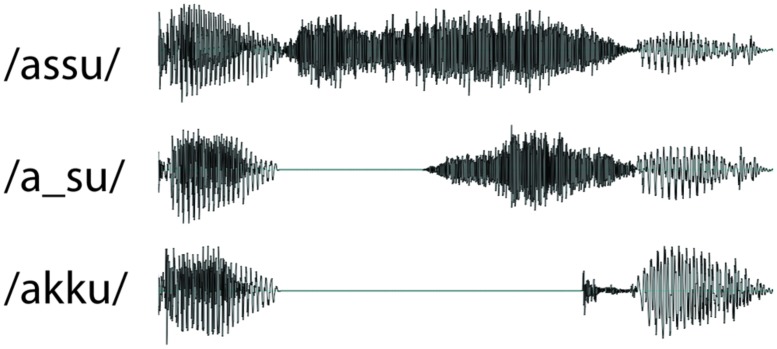
**Example waveforms of stimuli**.

Acoustically speaking, there are two types of Japanese obstruent geminate consonants, also known as ‘sokuon’: silent and fricative (Kawahara, in press). Silent geminates are produced by an abrupt suspension of articulator movements and/or by sustaining an oral closure or constriction for a short duration (e.g., stop geminate consonants, /kk/, /tt/). Fricative geminates contain sustained frication throughout their duration (e.g., /ss/). **Figure 1** (/assu/ and /akku/) illustrates these examples. The duration of the closure or sustained frication tends to align with the duration of a mora (Vance, 1987; Han, 1994), a perceptual unit that Japanese native listeners use (Otake et al., 1993, 1996; Cutler and Otake, 1994; Murty et al., 2007). Takahashi (1998) studied the frequency of occurrence of both types. He found that, among the 2,467 geminate consonants that appeared in the Iwanami Japanese dictionary (5th Edn), 76% were silent geminates and 24% were fricative geminates.

With regards to phonology, Japanese is one of the few languages that make use of moraic structure (Vance, 1987). The literature traditionally treats the first consonant of Japanese geminate consonants (‘sokuon’) as one mora duration of the voiceless obstruent /Q/ (Han, 1994), while the second consonant together with the following vowel are treated as a standard mora. However, there has been no clear agreement on whether and how Japanese native listeners make use of /Q/ when perceiving geminate consonants. For example, Tamaoka and Terao (2004) proposed that special morae (such as the moraic nasal /N/ and /Q/) are not processed as a single mora unit, but as a part of the previous unit (e.g., CVN-CV rather than CV-N-CV). Based on this, they suggested that the latency of naming a word with CVNCV structure (which should be represented in two units) should be shorter than that with CVCVCV structure (three units). However, results regarding the moraic obstruent /Q/ were inconclusive: the authors interpreted this as /Q/ being segmented in both ways (CV-Q-CV and CVQ-CV). The current study addresses the same issue but with the method that taps directly to characteristics of abstract representations of Japanese geminate consonants (see below sections for detail). If /Q/ is perceived by native Japanese listeners as a single unit mora, then /Q/ should represent the initial portion of geminates which, as mentioned previously, can be acoustically realized in two distinct ways (silent or fricative). This is a very different way of representing geminate consonants from, e.g., one long consonant (Kingston et al., 2009; Hardison and Motohashi-Saigo, 2010; Tagliapietra and McQueen, 2010).

From an orthographic perspective, the two types of geminates are partially represented by the same Japanese syllabic character 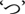 (a small ‘tsu’). For example, ‘shissaku’ (mistake) is written in four Japanese syllabic characters 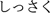 (shi-tsu-sa-ku) and ‘shikkaku’ (disqualified) is also written using the same character 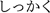 (shi-*tsu*-ka-ku). Similar to /Q/, as such, one character 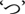 represents the first part of the geminate consonants while there are two acoustical types. In contrast, alphabetic languages, such as Italian, use double consonants to represent geminates: /ss/, e.g., Italian words ‘asso’ (ace) vs. /kk/, e.g., ‘rocca’ (fortress).

A discussion of the fundamental differences between phonological and orthographic systems among languages is beyond the scope of this paper. Instead, the current study asks how crucial it is for native listeners of Japanese to distinguish such acoustical differences when perceiving geminate consonants. Considering that neither the phonological nor the orthographic system suggest one-to-one correspondence between the representation and acoustic realizations – one representation (/Q/ or ) corresponds with two geminate types (silent and fricative ones) – we hypothesized that making perceptual distinctions between the two types of acoustic signals may not be essential for Japanese native listening. In other words, abstract representation of silent and fricative geminates may be similar for Japanese listeners. However, native speakers of other languages might treat these two as clearly separated perceptual categories.

We compared native listeners of Japanese and Dutch with regard to perception of spoken pseudo words containing either fricative geminates /ss/ or an artificial fricative geminate that consisted of a silence followed by a fricative singleton consonant /_/ + /s/ (Sadakata et al., 2012). This preliminary analysis revealed that these two stimuli indeed sounded similar for Japanese group, suggesting that it is not crucial for them to distinguish the fricative and silence geminate types, while these sounded less similar for Dutch native listeners. The results thus supported the hypothesis that Japanese make use of specific listening strategy: the two different types of geminates consonants were perceived in a similar way. However, because the Dutch language does not include geminate consonants in its inventory (Booij, 1995), the difference between Japanese and Dutch groups may simply reflect the presence or absence of geminates in the phoneme inventory of a given speaker’s language. In other words, there is a possibility that native speakers of all geminate languages, such as Italian and Finish, may rely on a similar strategy as Japanese one. Because our hypothesis is more specific to Japanese native listening, the current study additionally presents the results of these experiments for native speakers of Italian.

The Italian language also includes phonemic contrasts between geminates and singleton consonants. However, unlike Japanese, it makes use of alphabetic representations (Pickett et al., 2000; Tagliapietra and McQueen, 2010), and does not use morae, but rather syllables for speech segmentation (e.g., Tabossi et al., 2000). As for segmentation of geminate consonants, Italians may share part of the strategy with Japanese, namely that it segments geminated consonants in two parts rather than treating it as a long consonant. There is a long-standing debate on whether Italian geminates are segmented into two parts or not (Loporcaro, 1996; Bertinetto and Loporcaro, 2005). The traditional phonological representation as presented in, e.g., Loporcaro (1996) suggests that the two consonants in Italian geminates are split, with each part belonging to the syllables before and after the geminate, respectively (e.g., asso ‘ace’ is syllabified as /as.so/).

While it may sound similar to the Japanese two-part representation, this model has different consequences when segmenting /ss/ and /_s/. With this Italian two-part segmentation, /assu/ will be heard as /as/ + /su/ while /a_su/ will be heard as /a/ + /su/. In other words, Italian representations of two sounds (/ss/ and /_s/) are likely to be different. However, the first Italian representation (/as/ + /su/) cannot apply to Japanese native listening because Japanese phonotactics does not allow coda consonants (Dupoux et al., 1999; Dehaene-Lambertz et al., 2000). Our specific hypothesis for Japanese representation of geminate consonants is that, instead of forming a coda consonant, the first portion of voiceless geminate consonants may form an independent perceptual unit in Japanese representation that is similar to a silence. As a result, both of /assu/ and /a_su/ are represented as /a/ + /_/ + /su/. In summary, both Italian phonology and orthography differ from those of Japanese. We therefore expect the Italian group to respond similarly to the Dutch group. In order to make a direct comparison among the three language groups, we report on the data previously collected from Japanese and Dutch groups together with additional analyses.

Two perceptual experiments, as in Sadakata et al. (2012), are reported here: a multi-voice categorization task and a multi-voice discrimination task. Although both make use of multiple voices and are therefore expected to reflect abstract linguistic representations that are independent from acoustic characteristics, the two tasks are expected to shed light on different levels of perception. The multi-voice categorization task presents four standard stimuli followed by one test stimulus (AAAAB), where participants are required to judge whether A and B belong to the same category or not (Dehaene-Lambertz et al., 2000). For example, the A sound might be /assu/ while the B sound could be either /a_su/ or /assu/. Participants were not instructed about the use of information regarding the presence/absence of silence when making responses. In other words, this task tests whether a participant tends to make use of or ignore the difference between /assu/ and /a_su/. Using this task, Dehaene-Lambertz et al. (2000) were able to capture the influence of phonotactics on perception. It is also efficient in controlling for task difficulty across the three participant groups. Conventional categorization tests often require participants to remember the target categories during subsequent categorization trials (e.g., 2AFC). This may introduce an undesired bias, as non-native speakers of Japanese may have more difficulty in remembering target categories throughout the test session, due to the fact that native speakers of Japanese produced the materials. The present task reduces the risk of this problem by not requiring participants to remember target categories.

The multi-voice discrimination test employed a modified version of the four-interval two-alternative forced choice task (4I-2AFC, Gerrits and Schouten, 2004). In this task, one deviant stimulus and three standard stimuli were presented each trial. The deviant stimuli occurred in either the second or the third position (ABAA or AABA). Participants were required to indicate the position of deviant stimuli. One crucial difference from the original study in Gerrits and Schouten (2004) was that each of the four words was presented in a different voice (four voices per trial). This was done in order to match this task as closely as possible to the multi-voice categorization test, as well as to ensure that participants’ sensitivity to the presence of silence was tested, independently of other acoustic cues (e.g., pitch heights, loudness envelopes). In other words, the only constant factor to determine the correct response in this task was an absence/presence of silence. In contrast to the multi-voice categorization test, this task thus requires that participants rely on the presence/absence of silence in the speech signal for making correct responses. In this way, it tests whether or not participants are sensitive to the presence/absence of silence embedded in speech signals.

We expected the results of the discrimination test to show that all three groups are sensitive to the presence/absence of a silence embedded in a speech sequence. More importantly, we predicted that the Japanese group would tend to ignore the difference between the two acoustic types of geminates in the categorization task, while the other groups would rely on the acoustic information. Such results would highlight the different abstract representations of fricative geminate consonants used by native speakers of Japanese, Dutch and Italian.

## MATERIALS AND METHODS

### PARTICIPANTS

Three groups of 16 native speakers of Japanese, Italian and Dutch took part in the two tests. Japanese participants were students of Kumamoto University (6 males and 10 females, average of 21.3 years old), Italian participants were students of Trento University (four males and 12 females, average of 25.4 years old), and Dutch participants were students of Radboud University of Nijmegen (three males and 13 females, average of 20.9 years old). The experiments were conducted in the respective countries of each of the three participant groups. All Japanese participants had followed English lessons (average 8.1 years). Some had also learned other languages, such as German, French, and Chinese. None of Italian and Dutch participants had intensive exposure to Japanese speech. Thirteen Italian participants had followed English lessons (average 7 years). Some of them spoke additional languages: French, German, Spanish, Catalan, and Esperanto. All Dutch participants had followed English lessons (average 6.8 years), and had also learned additional languages, such as French, German, Spanish, and Chinese. Written consent was obtained from all participants prior to participation. The study was carried out in accordance with the Declaration of Helsinki and Dutch legislation. The research was approved by the research ethics committee of Behavioral Research, Radboud University Nijmegen, reference number 24092009.

#### Stimuli

**Figure 1** presents the waveforms of the experimental stimuli. Three types of stimuli were constructed with the following parameters: V1-/_s/-V2 (e.g., /a/ = 90 ms, /_/ = 100 ms, /s/ = 160 ms, /u/ = 100 ms), V1-/ss/-V2 (/a/ = 90 ms, /ss/ = 260 ms, /u/ = 100 ms), and V1-/kk/-V2 (/a/ = 90 ms, /_/ = 220 ms, /k/ = 40 ms, /u/ = 100 ms). These durations were chosen to correspond to the natural range of production (Sadakata and McQueen, 2013). Affricates (e.g., /ts/) are sometimes described as a plosive (similar to a silence) followed by a frication. However, the affricate /ts/ tends to be perceived when frication noise is much shorter (e.g., Repp et al., 1978). Therefore our /_s/ stimuli is, acoustically speaking, clearly distinct from both fricative and affricate geminates. Six combinations of V1 and V2 were chosen, leading to a total of 18 pseudo words (**Table 1**). Stimuli were created using six female voices and one male voice. The individual voices each corresponded to a set of recordings made by native speakers of Japanese. The speakers produced /VsV/, /VssV/, and /VkkV/ targets. Using each recording as a template, synthesized stimuli were created with identical interval durations for the three types of stimuli (/ss/, /_s/, and /kk/) using Praat (Boersma and Weenink, 2008). The pitch accent patterns of the two vowels /a/ and /u/ were high–low. Onsets and offsets of each element were ramped (5 ms) to avoid clipping at the concatenation points. All materials were recorded at a sample rate of 9.6 kHz and low-pass filtered at 5 kHz in order to eliminate the low-level background noise. Average sound levels were normalized to and presented at approximately 70 dB.

**Table 1:** List of pseudo words.

Stimulus type	Pseudo words
V1-/_s/-V2	a_su, i_se, u_sa, u_se, o_sa, o_su
V1-/ss/-V2	assu, isse, ussa, usse, ossa, ossu
V1-/kk/-V2	akku, ikke, ukka, ukke, okka, okku

### PROCEDURE

Participants took part in the multi-voice categorization test first, followed by the multi-voice discrimination test.

#### Multi-voice categorization test

The multi-voice categorization task was designed in the same manner as in Dehaene-Lambertz et al. (2000). Participants were presented with five words in each trial (ISI = 500 ms): four standard stimuli and one test stimulus (AAAAB). The first four items (standard) were either /ss/ or /_s/, presented by four different female voices that were randomly chosen from the six available voices (see the ‘Methods’ section for details about stimuli construction). The fifth item (test) was either /ss/, /_s/, or /kk/, spoken by a male voice. The use of the male voice for the test item is in accordance with the original study. We used this large difference in voice quality between the standard and the test stimuli because it helps participants to clearly distinguish between the standards and the test item. Participants were asked to judge whether the test (fifth) word was the same as the standard items. The three test stimuli conditions are referred to as *Same*, *Different,* and *Filler*, respectively (see **Table 2**). Each test condition was presented 120 times, resulting in 360 trials randomly divided in 10 blocks.

**Table 2:** Experiment conditions and predictions.

Condition	Standard-Test	Predicted response		
		JP	IT	NL
Same	/ss/ – /ss/	Same	Same	Same
	/_s/ – /_s/			
Different	/ss/ – /_s/	Same	Different	Different
	/_s/ – /ss/			
Filler	/ss/ – /kk/	Different	Different	Different
	/_s/ – /kk/			

#### Multi-voice discrimination test

The discrimination trial sequences consisted of four words: one deviant stimulus and three standard stimuli. The deviant stimuli occurred either in the second or the third position with a 50% a priori probability (ABAA or AABA). The task was to discriminate the deviant stimulus by indicating the position of its occurrence. Two conditions were created: Silent and Stop. For both conditions, /ss/ served as standards. The Silent condition included /_s/ and the Stop condition included /kk/ as a deviant. In each trial, four words were presented (Inter-stimulus interval ISI = 500 ms). Four different female voices from the six available voices were randomly chosen for each of four words within a trial. The Silent and Stop condition were each presented 45 times, resulting in 90 trials randomly divided into six blocks.

#### Apparatus

All sounds were recorded using a linear PCM recorder (Sony PCM – D1) with a sampling rate of 9.6 kHz in a sound attenuated booth. A DELL notebook computer with an Intel CoreDuo processer (4 GB RAM) was used to perform all experiments. Sony MDR-7506 headphones and a 15.4-inch TFT screen were used to present auditory stimuli and visual instructions, respectively. The average sound pressure level (SPL) of the headphones was adjusted to around 69 dB. The application Presentation (version 14.3, Neurobehavioral Systems) was used for presenting instructions and stimuli as well as for collecting responses. Participants responded using the computer keyboard (“s” and “:” buttons).

## RESULTS

### MULTI-VOICE CATEGORIZATION TEST

**FIGURE 2:**
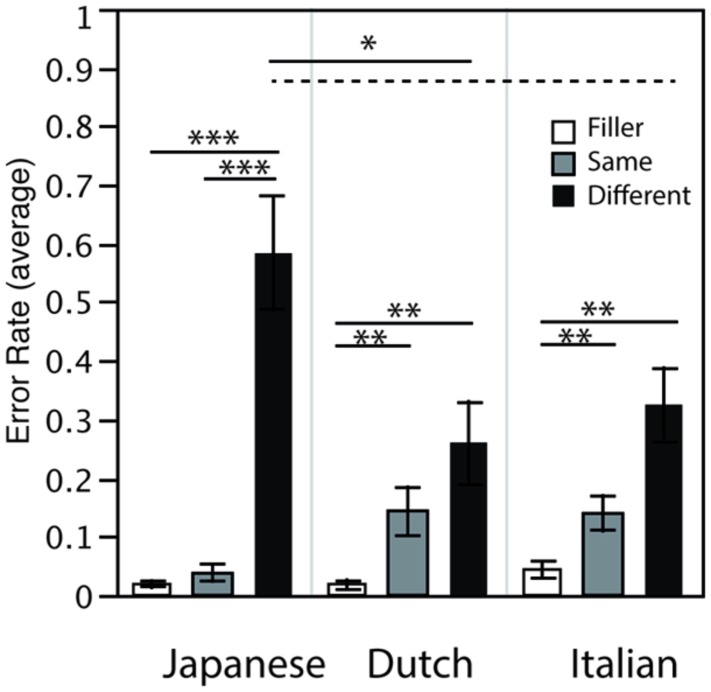
**Mean error rates of the multi-voice categorization task.** Results in all 3 conditions are indicated for Japanese, Dutch and Italian listeners (***p < 0.001, **p < 0.01, *p < 0.05, broken line *p* = 0.07).

A mixed model ANOVA with Condition (Same/Different/Filler) as a within-subjects factor and native-language (JP = Japanese, NL = Dutch, IT = Italian) as between-subjects factors on error rates indicated significant main effects of Condition [*F*(2,90) = 47.039; _GG_ = 0.609; *p* < 0.0001; $\eta_{p}^{2}$ = 0.511] as well as significant interaction between Condition and Native-language [*F*(4,90) = 6.3; _GG_ = 0.609; *p* < 0.01; $\eta_{p}^{2}$ = 0.220]. There was no significant effect of Native-language [*F*(2,45) = 1.718; n.s.]. Further analysis indicated several significant simple effects, which are discussed below and are indicated in **Figure 2**. Relatively low error rates for the Filler condition for all three groups indicate that all participants performed the task correctly. As expected, Japanese data indicated significantly higher error rates for the Different condition than for the Same and Filler conditions. The Different condition presented /ss/ and /_s/, and the higher error rates for this condition confirms the hypothesis that the silent (/_s/) and fricative (/ss/) stimuli sounded similar to Japanese ears: making distinctions of these two acoustic types was not crucial for them. In contrast, a different result pattern of Same, Different and Filler conditions was observed for Dutch and Italian data as compared to that for Japanese. Significantly lower error rates were observed for the Filler condition than for the other two conditions: crucially, the error rate for the Same and Different conditions did not significantly differ for them. It is also possible that relatively higher variance in the Same condition for Dutch and Italian groups resulted in non-significant difference between the Same and Different condition, potentially due to the use of Japanese speech sounds. Nevertheless, this suggests that native speakers of Dutch and Italian perceived and indicated the difference between the silent (/_s/) and fricative (/ss/) stimuli relatively well.

**FIGURE 3:**
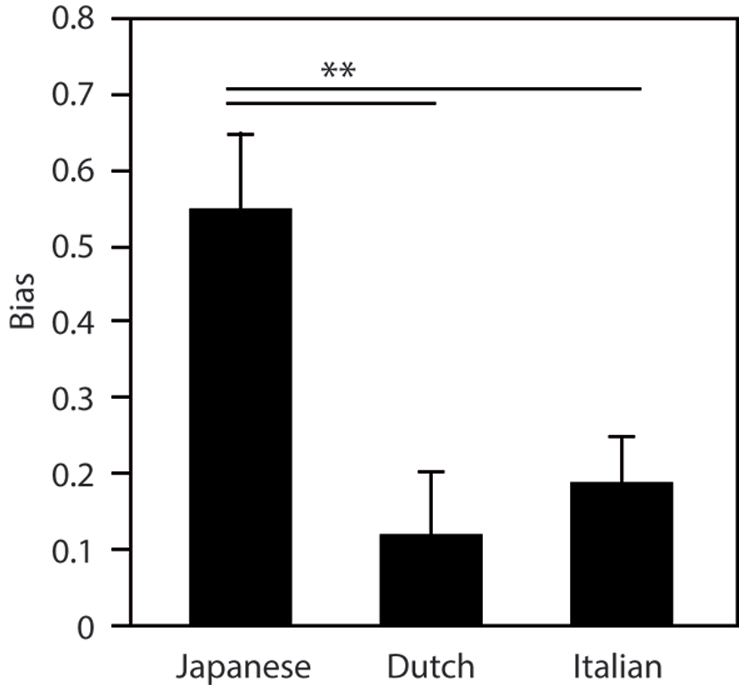
Bias measure, calculated as the difference between the error rates of the Different and Same conditions (Different – Same) for Japanese, Dutch and Italian listeners (**p < 0.01).

**Figure 3** presents a measure of bias, which is calculated as the difference between the error rates of the Different and Same conditions (Different – Same). Here, the error rate of the Same condition is used as an estimate of an individual’s task accuracy: the higher the error rate in this condition, the less accurate one is in performing the task. Therefore, subtracting the error rate of the Same condition from the Different condition indicates participants’ bias toward the same response for the Different condition. A one-way ANOVA with native-language (JP = Japanese, NL = Dutch, IT = Italian) as a between-groups factor on the bias measure indicated a strong significant main effect [*F*(2,47) = 7.69; *p* < 0.001; $\eta_{p}^{2}$ = 0.255). A multiple comparisons analysis indicated that the Japanese group showed significantly greater bias than both the Italian group and the Dutch group (Tukey, both *p* < 0.01). This indicates that Japanese group had a stronger tendency toward “same” responses than the other two groups when presented with /_s/ or /ss/.

### MULTI-VOICE DISCRIMINATION TEST

Error rates for the discrimination test are summarized in **Table 3**. A two-way mixed-model ANOVA with Condition (Silence/Stop) as a within-subjects factor and Native-language (JP/NL/IT) as a between-subjects factor on the Error rate indicated a significant effect of Condition [*F*(1,45) = 26.41; *p* < 0.0001; $\eta_{p}^{2}$ = 0.37] with higher error rates for the Silence than the Stop condition. Neither the main effect of Native-language nor interaction effect was significant [*F*(2,45) = 0.179 n.s. and *F*(1,45) = 0.7436 n.s., respectively]. The results indicated that the Silence stimuli /a_su/ were more difficult to discriminate from /assu/ than the Stop stimuli /akku/ for all participants. Nevertheless, the error rates for the Silence condition were lower than 10%, suggesting that participants performed the task fairly well. Crucially, there was no difference in this sensitivity among native speakers of Japanese, Dutch and Italian.

**Table 3:** Error rates (%) of the discrimination tests.

Condition	Standard – Deviant	JP	IT	NL
Silence	/assu/ – /a_su/	9.3 (8.8)	7.5 (8.2)	9.6 (14)
Stop	/assu/ – /akku/	0.6 (1.3)	2.8 (4.8)	2.9 (5.1)

*Numbers in parentheses indicate SD, JP: Japanese, IT: Italian, NL: Dutch*.

## DISCUSSION

The current study tested whether the acoustic difference between /ss/ and /_s/ is perceptually important for Japanese native listening. Acoustically speaking, /_s/ is closer to /ss/ than /kk/. Therefore it is not too surprising if /_s/ is perceived as more similar to /ss/ than to /kk/. However, Japanese native listeners tended to categorize /ss/ and /_s/ into the same group more often than Dutch native listeners did (Sadakata et al., 2012). The new analysis in this study (**Figure 3**) clearly indicated that there is a stronger “same” bias of Japanese group than Dutch group. The critical questions are, then, what do these results suggest and why such a specific way of listening came to exist. We hypothesized that this same bias is specific to Japanese native listening that is related to how Japanese language represent geminate consonants. However, the difference between Japanese and Dutch groups could simply be explained by the presence/absence of geminate consonants in participants’ native language, as the Dutch language does not include geminate consonants while Japanese does. In order to disentangle this, a third group of participants – native Italian listeners – was included here because Italian speech includes geminate consonants, but the manner in which Italian phonology and orthography represent geminate consonants deviates from that of Japanese (Pickett et al., 2000; Tagliapietra and McQueen, 2010). Our results indicated that the Dutch and Italian groups exhibited similar patterns, namely that they could more clearly distinguish the critical geminate sounds than the native Japanese listeners. This strengthens the idea that the observed tendency is specific to Japanese native listening: the minimal pairs of fricative and silent geminate pseudowords used as stimuli in the experiment were perceptually similar for Japanese native listeners, but not for the other two groups. The multi-voice discrimination test confirmed that the Japanese group was able to distinguish silence from frication when they were required to pay attention to the difference. Taken together, we think that the acoustic realization of the initial portion of the geminate consonants is not critical for Japanese native listening. In fact, the insertion of a silence (/_s/), which is acoustically highly unlikely, tended to be accepted as a normal fricative geminate consonant substantially more often by the Japanese participants than the Italian and Dutch participants. This can be explained if we assume that the Japanese abstract representation of geminate consonants, notably the first portion of it, is somewhat similar to a silence.

It is interesting to note that, using the same multi-voice sequence, the two experiments tapped different stages of linguistic processing. One crucial difference between the tasks is that the multi-voice categorization task does not force listeners to use the presence/absence of silence for making judgments while the multi-voice discrimination experiment does by requiring listeners to identify stimuli that differ from the others with respect to the presence or absence of silence. Another important difference, which may have played a role, is the level of attention level required by the two tasks. Fujisaki and Kawashima (1971) put forward a model with regard to speech perception that incorporates two listening modes, one that relies on categorical phonemic judgments and another based on short-term auditory memory representations of acoustic information. It is well known that traditional perceptual discrimination tasks (such as AX and ABX task) reflect phonemic categorization process in responses. However, by placing two flankers before and after the AX task, Gerrits and Schouten (2004) succeeded in creating a new discrimination task, namely 4I2AFC, which completely eliminated the influence of phonemic categorization on discrimination judgment; listeners in the study relied exclusively on short-term auditory memory for discrimination. The current study suggested that, even though the required judgments were not purely based on acoustic attributes of the stimuli, the multi-voice 4IAFC task seemed to induce listeners to utilize short-term auditory memory representations of acoustic information. The detection of the “odd-one-out” relied more on comparison of stimuli that were presented before/after the target word in the multi-voice discrimination (4IAFC) task, while the judgments in the multi-voice categorization task seemed to reflect more on abstract representations.

The tendency that Japanese listeners exhibit in the current study is compatible with both Japanese phonology and orthography. In both systems, one representation is used to characterize the first part of geminate consonants despite the acoustic difference between fricatives and stop geminate consonants: the voiceless obstruent /Q/ (Vance, 1987; Han, 1994) in phonology and the special character 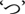 in orthography. Although it is impossible to address causal relationships in this study, we believe it is still worthwhile to discuss how the relation among these systems and the observed perceptual effect could be disentangled. Using the same multi-voice categorization task with EEG measurements, Dehaene-Lambertz et al. (2000) demonstrated that the influence of phonotactics seems to take place at early perceptual stage of processing, namely that the phonotactic constraints are already available when our system parses and segments ongoing acoustic information. In contrast to this, the orthographic influence seems to influence later stages of processing. Ventura et al. (2004) and Pattamadilok et al. (2007) found that the effect of orthographic information processing on perceptual responses disappears when the task does not require lexical access. One approach to extend the current discussion would be to measure neural correlates associated with our geminate consonant stimuli and investigate the time course of processing differences among listener groups.

It has been suggested that moraic segmentation strategies in Japanese listeners develop together with the acquisition of literacy (Inagaki et al., 2000). However, this same study reported that the acquisition of literacy did not increase a listener’s bias toward moraic segmentation of geminate consonants: surprisingly, 6-years old children indicated bias toward syllabic segmentation. A shift toward moraic segmentation may take place for geminate consonants too, as has been shown for the moraic nasal /N/, but at a later stage. Tamaoka and Terao (2004) reported that voiceless moraic obstruent /Q/ is treated either as an independent unit (CV-Q-CV) or as a part of the previous mora (CVQ-CV). These studies indicate that representations of special morae, such as /N/ and /Q/ do not develop in the same manner. In particular, the nature of the geminate consonant representations utilized by native Japanese listeners during speech processing needs further investigation. The present multi-voice categorization experiment paradigm may be able to contribute to this by asking whether learning to write 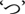 supports the formation of a common abstract representation of geminate consonants. If so, then children may show different patterns of perception before and after learning to write 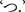 with the former producing results more along the lines of those observed for the Italian and Dutch groups in this experiment.

To summarize, the current study highlighted a new case in which Japanese native listeners exhibit a specific listening tendency with respect to perception of Japanese geminate consonants that is similar to how Japanese phonology and orthography represent geminate consonants. Unlike previous studies that were inconclusive with respect to the manner in which native speakers segment geminate consonants, our study demonstrated a relatively strong effect of Japanese-specific listening. Our experimental methods may thus offer a new path to investigate the relationship between development of orthography and speech perception.

## Conflict of Interest Statement

The authors declare that the research was conducted in the absence of any commercial or financial relationships that could be construed as a potential conflict of interest.
